# m6A-Related lncRNA to Develop Prognostic Signature and Predict the Immune Landscape in Bladder Cancer

**DOI:** 10.1155/2021/7488188

**Published:** 2021-07-24

**Authors:** Zuwei Li, Yuwu Li, Weizhang Zhong, Peiyuan Huang

**Affiliations:** ^1^Department of Urology, Gaozhou People's Hospital, Maoming 525200, China; ^2^Department of Pharmacy, Gaozhou People's Hospital, Maoming 525200, China

## Abstract

Abnormal m6A methylation plays a significant role in cancer progression. Increasingly, researchers have focused on developing lncRNA signatures to evaluate the prognosis of cancer patients. The specific function of m6A-related lncRNAs in the prognosis of bladder cancer patients and the immune microenvironment of bladder cancer remains elusive. Herein, we performed a comprehensive analysis of m6A-related lncRNA prognostic values and their association with the immune microenvironment in bladder cancer using the TCGA dataset. A total of 9 m6A-related lncRNAs were dramatically correlated with overall survival outcomes in bladder cancer. Two molecular subtypes (cluster 1 and cluster 2) were identified by consensus clustering for 9 m6A-related prognostic lncRNAs. Cluster 1 was significantly correlated with poor prognosis, advanced clinical stage, higher PD-L1 expression, a higher ESTIMATEScore and immuneScore, and distinct immune cell infiltration. GSEA revealed the enrichment of apoptosis and the JAK-STAT signaling pathway in cluster 2. A prognostic risk score was constructed using 9 m6A-related prognostic lncRNAs, which functioned as an independent prognostic factor for bladder cancer. Moreover, bladder cancer patients in the low-risk score group had a higher pN stage, pT stage, and clinical stage and a lower tumor grade and immuneScore. The risk score was correlated with the infiltration levels of certain immune cells, including B cells, plasma cells, follicular helper T cells, regulatory T cells, resting NK cells, neutrophils, M0 macrophages, M1 macrophages, and M2 macrophages. Collectively, our study elucidated the important role of m6A-related lncRNAs in the prognosis of bladder cancer patients and in the bladder cancer immune microenvironment. The results suggest that the components of the m6A-related prognostic lncRNA signature might serve as a crucial mediator of the immune microenvironment in bladder cancer, representing promising therapeutic targets for improving immunotherapeutic efficacy.

## 1. Background

Bladder cancer (BC) is one of the most frequent urinary malignancies in China and has a high incidence rate [1]. Transitional cell carcinoma ranks approximately 90% of bladder cancer. Most of the BCs are nonmuscle invasive (MI) BCs, which readily relapse and develop into muscle invasive bladder cancers [2]. Approximately 30% of bladder cancers are MIBCs, whose gold standard for their treatment is radical cystectomy and pelvic lymph node dissection [3]. The prognosis of MIBC patients is poor [4]. Although several clinical features and molecular biomarkers have been applied for the prognosis of bladder cancer patients, these approaches are all limited to some extent. Thus, it is necessary to construct a new predictive model and identify new prognostic markers for bladder cancer.

N6-methylandenosine (m6A) modification, one of the most common epigenetic methylation modifications, plays an important part in many biological processes, including RNA splicing, export, stability, and translation [5]. m6A methylation is significantly related to the levels of intracellular methyltransferases (“writers”) and demethylases (“erasers”), while binding proteins (“readers”) interact with m6A methylation sites to perform several biological functions [6]. Abnormal m6A methylation is involved in the progression of cancer by regulating various biological processes, including cell differentiation, immunoreaction, and miRNA editing [7]. Moreover, certain regulators of m6A methylation serve as prognosis biomarkers for certain cancers. For example, YTHDF1 and HNRNPC were suggested to be prognostic biomarkers of colon cancer [8]. Another study revealed that HNRNPC is a marker for prognosis in glioblastoma multiforme and contributes to carcinogenesis [9]. Another bioinformatics study revealed that the regulators of m6A methylation could promote tumor progression and affect the overall survival (OS) of BC patients [10].

Long noncoding RNAs (lncRNAs), a category of RNAs with transcript lengths over 200 nucleotides, regulate 70% of gene expression in mammals by interacting with DNA, RNA, and proteins [11]. Aberrant lncRNA expression is associated with the regulation of the proliferation, invasion, and apoptosis of tumor cell, thus affecting the pathogenicity of bladder cancer and patient prognosis. The lncRNA LINC00641 has been shown to act as a prognostic biomarker and to inhibit bladder cancer progression [12]. Another study revealed that the lncRNA CCAT1 could promote tumor cell biological progression in bladder cancer [13]. However, the overall functions of m6A methylation-related lncRNAs in cancers remain a mystery. Therefore, exploring m6A methylation-related lncRNAs and identifying prognostic biomarkers among these lncRNAs are of significance.

Based on The Cancer Genome Atlas (TCGA) database, the identification of prognostic gene signatures has become possible [14]. Using the lncRNA expression profiles of TCGA bladder cancer cohort, we identified prognostic biomarkers based on m6A methylation-related lncRNAs and constructed a prognostic model for bladder cancer by using bioinformatics methods. We also evaluated the correlation between the signature lncRNAs and immune infiltration in bladder cancer.

## 2. Materials and Methods

### 2.1. Datasets

The mRNA expression profiles of BC patients were isolated from the TCGA database, and we obtained data on 412 BC tissues and 19 normal bladder tissues. Moreover, the clinical characteristics of the BC patients, including age, grade, and survival status, were obtained from the TCGA.

### 2.2. Identification of m6A Methylation-Related lncRNA

According to previous publications, a total of 23 m6A RNA methylation regulators (METTL3, METTL14, METTL16, WTAP, VIRMA, ZC3H13, RBM15, RBM15B, YTHDC1, YTHDC2, YTHDF1, YTHDF2, YTHDF3, HNRNPC, FMR1, LRPPRC, HNRNPA2B1, IGF2BP1, IGF2BP2, IGF2BP3, RBMX, FT0, and ALKBH5) were obtained [15, 16]. lncRNAs were defined using the long noncoding RNA annotation file of the GENCODE website. Based on the ensemble IDs of the genes, a total of 14087 lncRNAs were identified in TCGA BC cohort. In order to obtain the expression level of lncRNA and m6A methylation regulators, we constructed a coexpression network using the “igraph” packages in the R program with |Pearson *R*| > 0.3 and *p* < 0.01. And those lncRNAs showed significant correlation with m6A methylation regulators and were determined to be m6A methylation-related lncRNAs.

### 2.3. Identification of Prognosis-Related lncRNA

We then explored the prognostic value of m6A-related lncRNAs using the “survival” package with a *p* value of 0.0001. We then generated a forest plot according to the data of the univariate Cox analysis. Moreover, we also constructed a heatmap using the “pheatmap” package to show the expression of those lncRNAs with significant prognosis value in BC tissues and normal tissues.

### 2.4. Bioinformatics Analysis

m6A methylation-related lncRNAs with significant prognostic value were selected for further analysis. We then classified bladder cancer into different subtypes using the “ConsensusClusterPlus” package (1,000 iterations and resampling rate of 80%). The gene expression patterns between each subtype were evaluated and visualized with the “pheatmap” package. Gene set enrichment analysis (GSEA) was conducted to detect the function of each bladder cancer subtype with a simulation of 500 and FDR of 0.05.

The ESTIMATE algorithm was utilized to calculate the immuneScore, stromalScore, and ESTIMATEScore for each bladder cancer patient using the “estimate” package. The infiltration abundance of 22 immune cell types in each BC subtype was visualized with the “vioplot” package.

The prognostic signature of m6A methylation-related lncRNAs was developed by LASSO regression analysis. By LASSO regression analysis, the coefficients of each bladder cancer case were calculated with the following computational equation: risk score = sum of coefficients × the lncRNA expression. The risk score of all the BC patients was computed in the training and test cohorts. Subsequently, the patients were separated into high- and low-risk groups with the cutoff point set as the median value of the risk score. Moreover, the correlation between the risk score and the abundance of immune cells was also calculated and “ggplot2” was used to visualize the result.

### 2.5. Statistical Analysis

All statistical tests were conducted with R version 4.0.1. The Mann–Whitney *U* test was applied to explore the mRNA levels of m6A methylation-related lncRNAs. The differences between two subgroups were evaluated with Student's *t*-test. The chi-square test was performed to compare categorical variables in the training and test cohorts. Survival curves were drawn using the Kaplan–Meier method. Pearson correlation tests were performed to explore the correlation among subtypes, clinicopathological features, risk scores, immune checkpoints expression, and immune infiltration levels. Univariate and multivariate analyses were performed with Cox regression models to explore the independent prognostic value of the risk scores integrated with other clinical features. *p* < 0.05 indicates statistical significance.

## 3. Results

### 3.1. Identification of m6A-Related lncRNAs in Bladder Cancer

**Figure 1:**
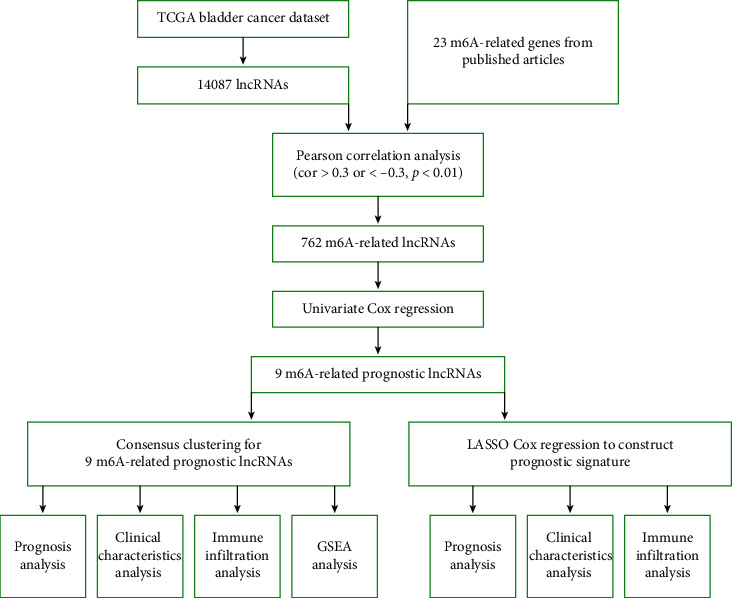
The work flow of the current study.

**Figure 2:**
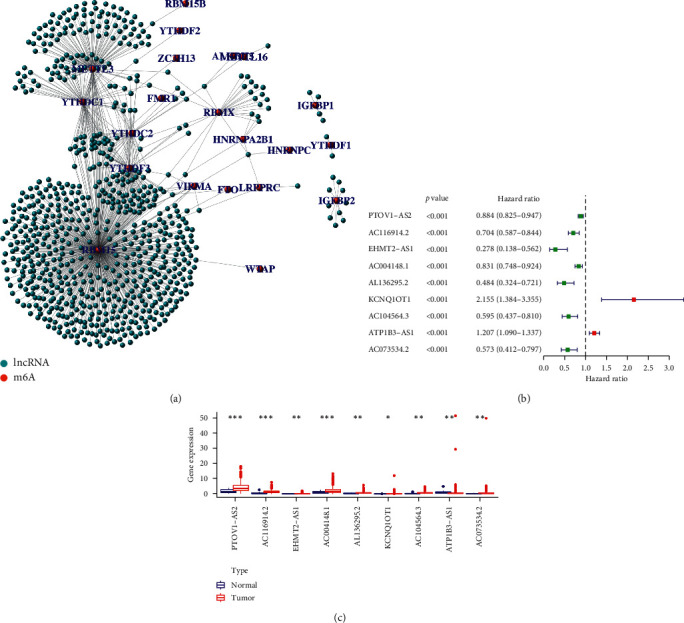
Identification of m6A-related prognostic lncRNAs in bladder cancer. (a) The coexpression network revealed m6A-related lncRNAs in bladder cancer. The orange dots represent m6A RNA methylation regulators and the green dots represent m6A-related lncRNAs. (b) A forest map showed 9 m6A-related prognostic lncRNAs identified by Cox proportional hazard regression. (c) Heatmap of the expression levels of 9 m6A-related prognostic lncRNAs in bladder cancer and adjacent normal tissues. ^*∗*^*p* < 0.05, ^*∗∗*^*p* < 0.01, and ^*∗∗∗*^*p* < 0.001.

Figure 1 displays the workflow of the current study. The clinical characteristics of bladder cancer patients were downloaded from the TCGA database and rearranged. A total of 412 cases of TCGA bladder cancer were obtained, and the clinical characteristics are shown in Table 1. Based on the lncRNA annotation file in GENCODE, we identified 14087 lncRNAs in the TCGA bladder cancer dataset. After extracting the expression profiles of 23 m6A methylation regulators in TCGA bladder cancer dataset, we evaluated the association between 23 m6A methylation regulators and 14087 lncRNAs. lncRNAs that were associated with one or more of the 23 m6A methylation regulators (|Pearson *R*| > 0.3 and *p* < 0.01) were defined as m6A-related lncRNAs. As a result, we obtained 762 m6A-related lncRNAs. The coexpression network between 23 m6A methylation regulators and 762 m6A-related lncRNAs is presented in Figure 2(a). Based on the prognostic value of these m6A-related lncRNAs, a univariate Cox regression analysis was performed to identify m6A-related prognostic lncRNAs with a *p* value of 0.0001. The data revealed that 9 m6A-related lncRNAs were markedly associated with OS in bladder cancer patients (Figure 2(b) and Table 2). We then analyzed the mRNA level of the 9 m6A-related prognostic lncRNAs in bladder cancer, which indicated the mRNA level of 8 m6A-related prognostic lncRNAs (PTOV1-AS2, AC116914.2, EHMT2-AS1, AC004148.1, AL136295.2, KCNQ1OT1, AC104564.3, and AC073534.2) was upregulated and the expression of 1 m6A-related prognostic lncRNA (ATP1B3-AS1) was decreased in BC tissues compared with their expression levels in bladder tissues (Figure 2(c)).

### 3.2. Consensus Clustering Categorized Patients according to m6A-Related Prognostic lncRNAs

**Figure 3:**
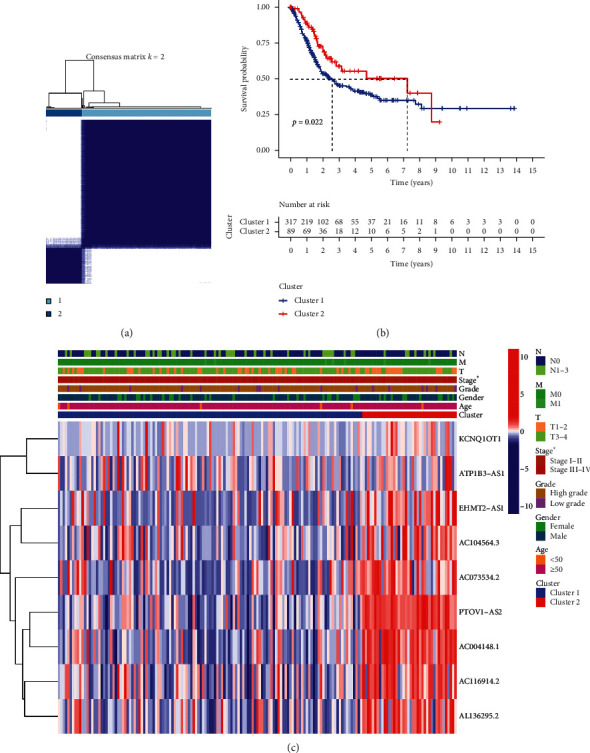
Consensus clustering of m6A-related prognostic lncRNAs. (a) TCGA bladder cancer cohorts were grouped into two clusters according to the consensus clustering matrix (*k* = 2). (b) Overall survival analysis revealed a better overall survival of bladder cancer patients in cluster 2 compared with that in cluster 1. (c) The heatmap of the two clusters along with clinicopathological characteristics.

Consensus clustering was utilized to separate bladder cancer patients into subgroups according to the expression of m6A-related prognostic lncRNAs. *k* = 2 was found to be optimal clustering stability from *k* = 2 to 9 based on the similarity displayed by the expression levels of m6A-related prognostic lncRNAs (Figure 3(a)). The cumulative distribution function, increment in the AUC, and tracking plot of subgroups for *k* = 2–9 are presented in Supplementary Figures 1B and 1C, respectively. A total of 406 bladder cancer patients were separated into cluster 1 and cluster 2 (Figure 3(a)). The OS rate of bladder cancer patients in cluster 1 was worse than that of those in cluster 2 (Figure 3(b), *p*=0.022). Moreover, we found that cluster 1 was markedly related to an advanced clinical stage (Figure 3(c), *p* < 0.05).

### 3.3. Consensus Clustering Correlated with Immune Infiltration

**Figure 4:**
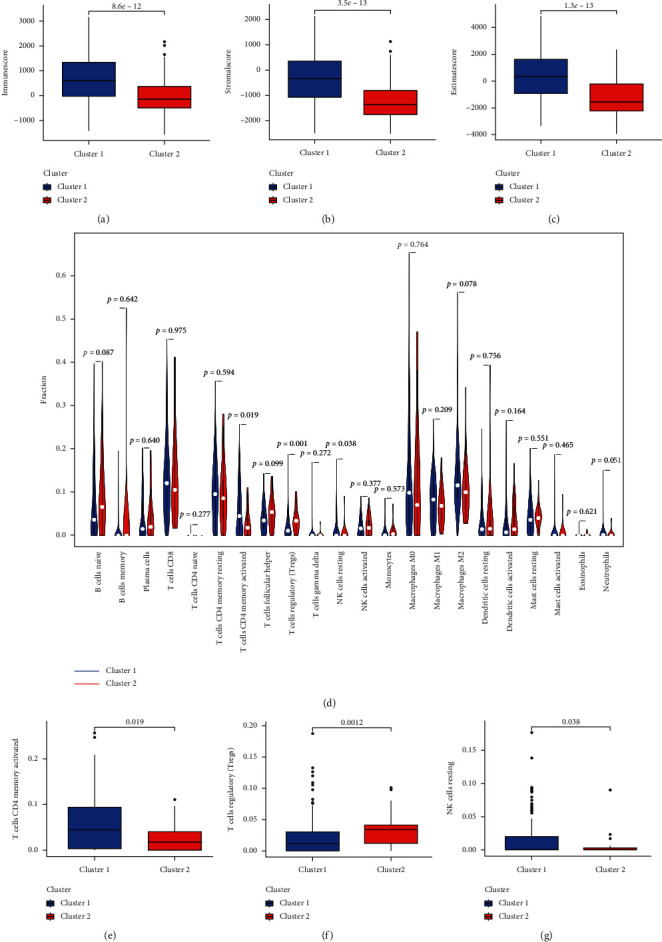
The correlation between two clusters and immune cell infiltration in bladder cancer. (a–c) Bladder cancer patients in cluster 1 had a higher immuneScore, stromalScore, and ESTIMATEScore compared with those in cluster 2. (d) The infiltrating levels of 22 immune cell types in cluster1/2 subtypes in the bladder cancer. (e–g) The infiltrating levels of the CD4 memory-activated T cells, regulatory T cells, and NK resting cells in the cluster1/2 subtypes.

**Figure 5:**
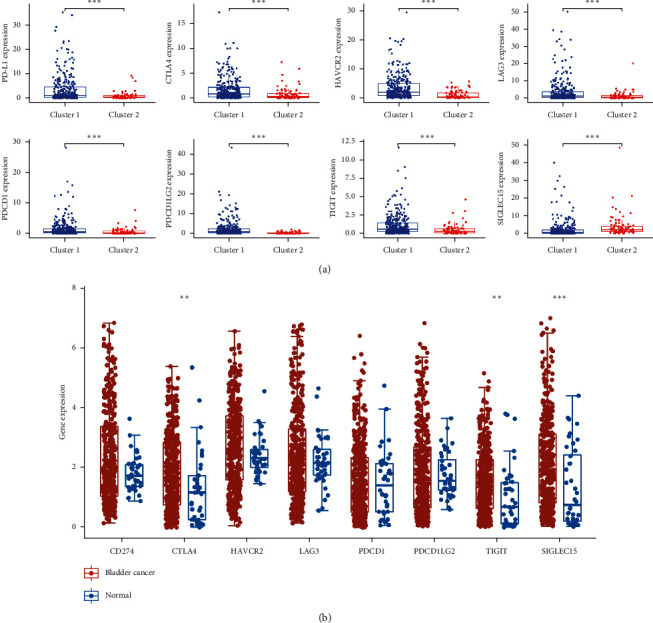
The expression of immune checkpoints in bladder cancer. (a) The mRNA level of PD-L1, CTLA4, HAVCR2, LAG3, PDCD1, PDCD1LG2, TIGIT, and SIGLEC15 in cluster 1 and cluster 2 of bladder cancer. (b) The mRNA level of CTLA4, TIGIT, and SIGLEC15 is upregulated in bladder cancer compared with normal tissues. ^*∗∗*^*p* < 0.01 and ^*∗∗∗*^*p* < 0.001.

To explore the role of m6A-related prognostic lncRNAs in the bladder cancer immune microenvironment, we then analyzed the difference in the immuneScore and immune cell infiltration level between cluster 1 and cluster 2. The average immuneScore (Figure 4(a), *p*=8.5*e*^−12^), stromalScore (Figure 4(b), *p*=3.5*e*^−13^), and ESTIMATEScore (Figure 4(c), *p*=1.3*e*^−13^) were higher in cluster 1 than in cluster 2. The infiltration abundance of 22 types of immune cells in each cluster is shown in Figure 4(d). As shown in Figures 4(e)–4(g), cluster 1 had a higher abundance of CD4 memory-activated T cells (*p*=0.019), a lower abundance of regulatory T cells (*p*=0.0012), and NK resting cells (*p*=0.038) compared to cluster 2. Moreover, we also found a positive correlation between macrophage M1 and macrophage M2 (Supplementary Figure 2). We then detected the mRNA level of immune checkpoints in each subtype and their correlation with m6A-related prognostic lncRNAs. The expression of PD-L1, CTLA4, HAVCR2, LAG3, PDCD1, PDCD1LG2, and TIGIT was higher while SIGLEC15 expression was significantly lower in cluster 1 than in cluster 2 (Figure 5(a), *p* < 0.001). We also found that the expression of CTLA4, TIGIT, and SIGLEC15 in BC tissues was significantly elevated (Figure 5(b), *p* < 0.001). Interestingly, the expression of PD-L1 (Supplementary Figure 3A), CTLA4 (Supplementary Figure 3B), HAVCR2 (Supplementary Figure 3C), LAG3 (Supplementary Figure 3D), and PDCD1LG2 (Supplementary Figure 4B) was significantly negatively associated with the expression of PTOV1-AS2, EHMT2-AS1, AC004148.1, and AC104564.3 (*p* < 0.05).

### 3.4. Enrichment Analysis of Each Bladder Cancer Subtype

GSEA was conducted to clarify the potential regulatory mechanisms leading to the differences between the two clusters of bladder cancer patients. Some cancer-related hallmarks, including the spliceosome and the mTOR and Notch signaling pathways, were significantly associated with cluster 1 (Supplementary Figure 5). Apoptosis and the chemokine, Toll-like receptor, and JAK-STAT signaling pathways were associated with cluster 2 (Supplementary Figure 6).

### 3.5. Construction of Prognostic Signature

**Figure 6:**
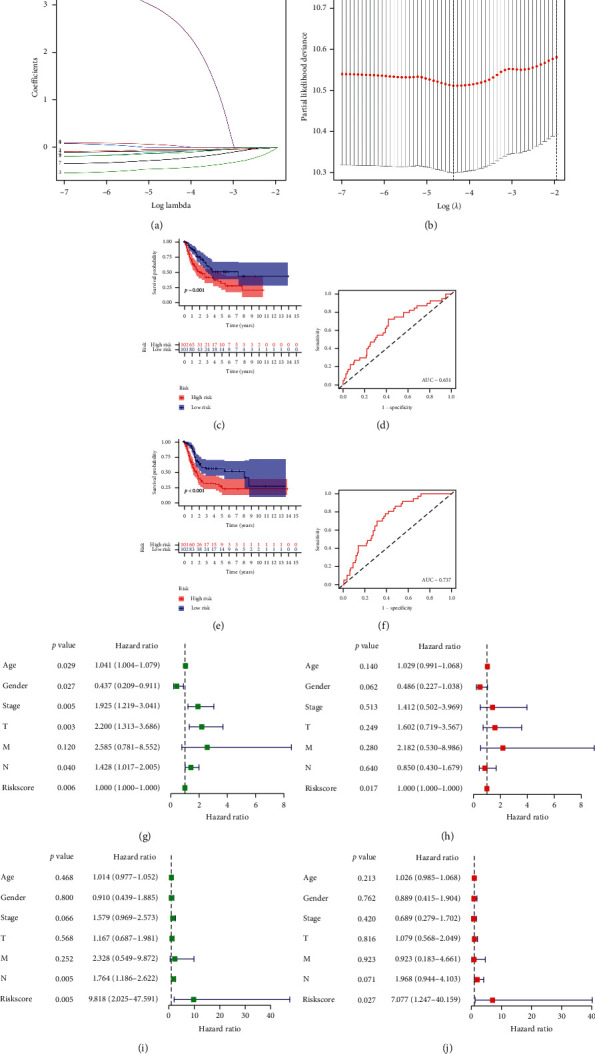
Construction the risk model of m6A-related prognostic lncRNAs in bladder cancer. (a) LASSO regression of 9 m6A-related prognostic lncRNAs. (b) Cross-validation for tuning the parameter selection in the LASSO regression. (c–f) Overall survival analysis for patients in high/low risk and the ROC curve of measuring the predictive value in training cohort (c–d) and test cohort (e–f). (g–j) Univariate and multivariate Cox hazard ratio analysis considering riskScore and age, gender, clinical stage, pT stage, pN stage, and pM stage in training cohort (g–h) and test cohort (i–j).

LASSO Cox analysis was applied to construct the prognostic signature using the 9 identified m6A-related prognostic lncRNAs. The coefficient and partial likelihood deviance of prognostic signature are shown in Figures 6(a) and 6(b). The 406 bladder cancer patients were randomly separated to training cohort and test cohort. The risk score of each patients was calculated using the following equation: risk score = (2.66 × KCNQ1OT1 expression) − (0.075 × PTOV1-AS2 expression) − (0.049 × AC116914.2 expression) − (0.439 × EHMT2-AS1 expression) − (0.094 × AL136295.2 expression) − (0.245 × AC104564.3 expression) + (0.036 × ATP1B3-AS1 expression) − (0.117 × AC073534.2 expression). The TCGA bladder cancer patients were separated into high-risk and low-risk groups. And OS curve suggested a poor prognosis in patients in the high-risk group compared with those in the low-risk group in both the training cohort (Figure 6(c)) and test cohort (Figure 6(e)), with an AUC of 0.651 in the training cohort (Figure 6(d)) and 0.737 in the test cohort (Figure 6(f)), indicating that the signatures comprising 9 m6A-related prognostic lncRNAs had a favorable discrimination performance in predicting bladder cancer patient prognosis.

To further explore the factors affecting the prognosis of bladder cancer, we then performed univariate and multivariate Cox regression analyses. The univariate Cox regression analysis showed that age, sex, clinical stage, pT stage, pN stage, and risk score were related to the prognosis of bladder cancer patients in the training cohort (Figure 6(g), all *p* < 0.05). Moreover, the multivariate Cox regression analysis revealed that the risk score (*p* < 0.001) was still significantly associated with the prognosis of bladder cancer patients (Figure 6(h)). In the test cohort, the univariate and multivariate Cox regression analyses were also performed, revealing the risk score as the factor affecting the prognosis of bladder cancer patients (Figures 6(i) and 6(j)).

**Figure 7:**
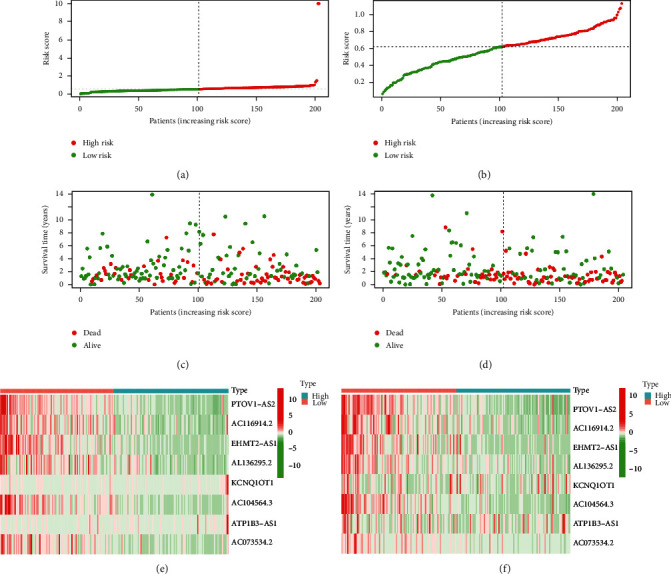
The risk model of m6A-related prognostic lncRNAs in bladder cancer. (a–b) Distribution of risk score of bladder cancer patients in training cohort and test cohort. (c–d) Survival status of each patient in training cohort and test cohort. (e–f) Heatmap about the expression of m6A-related prognostic lncRNAs in training cohort and test cohort.

Figure 7 shows the risk score distribution (Figures 7(a) and 7(b)) and survival status (Figures 7(c) and 7(d)) of each BC patient in the training and test cohorts. The mRNA levels of lncRNA, including KCNQ1OT1 and ATP1B3-AS1, were downregulated in the high-risk group, whereas protective m6A-related lncRNAs, including PTOV1-AS2, AC116914.2, EHMT2-AS1, AL136295.2, AC104564.3, and AC073534.2, were expressed at low levels in the high-risk group in both the bladder cancer training and test cohorts (Figures 7(e) and 7(f)).

We then verified the prognosis of the risk score in different groups of BC patients, which found that the high-risk group had a poor prognosis compared with the low-risk group in bladder cancer patients aged >50 years (Supplementary Figure 7A, *p* < 0.01) and male and female bladder cancer patients (Supplementary Figure 7B, all *p* < 0.05). Moreover, the high-risk group had a poor prognosis compared with the low-risk group in bladder cancer patients with a high tumor grade (Supplementary Figure 7C, *p* < 0.01), pT3-4 stage (Supplementary Figure 8A, *p*=0.004), pM0 stage (Supplementary Figure 8B, *p* < 0.01), pN0 stage (Supplementary Figure 8C, *p*=0.015), and in those at an advanced clinical stage (Supplementary Figure 9A, *p*=0.009).

### 3.6. Risk Score Associated with Clinical Characteristics

**Figure 8:**
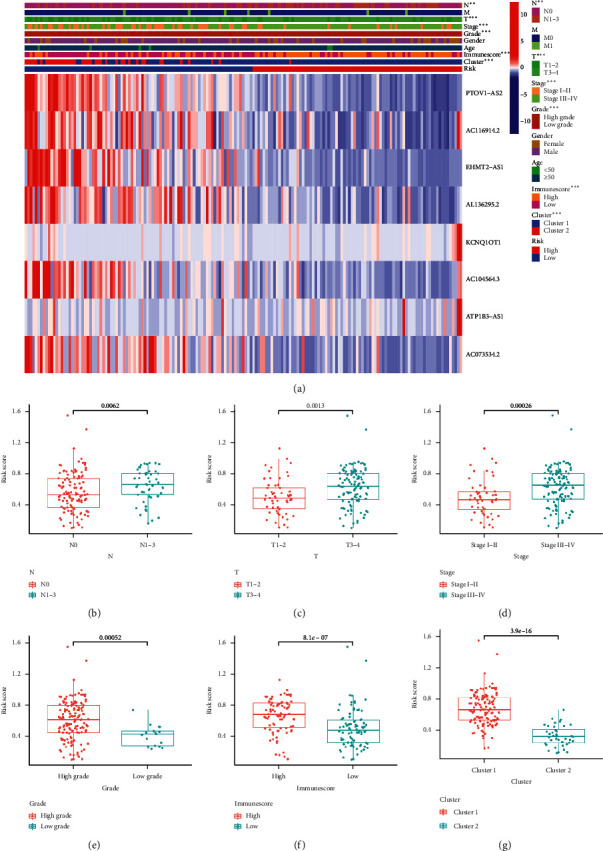
The risk score correlated with clinicopathological features and immuneScore in bladder cancer. (a) Heatmap revealed a significant pN stage, pT stage, clinical stage, tumor grade, immuneScore, and cluster between high-risk and low-risk groups. (b–g) The risk score in different pN stage (b), pT stage (c), clinical stage (d), tumor grade (e), immuneScore (f), and cluster (g) of bladder cancer patients. ^*∗*^*p* < 0.05, ^*∗∗*^*p* < 0.01, and ^*∗∗∗*^*p* < 0.001.

The heatmap in Figure 8(a) revealed the mRNA level of lncRNAs in the high- and low-risk groups in TCGA BC cohort. The results suggested that the expression levels of KCNQ1OT1 and ATP1B3-AS1 were higher in the high-risk group, whereas the expression levels of PTOV1-AS2, AC116914.2, EHMT2-AS1, AL136295.2, AC104564.3, and AC073534.2 were lower in the high-risk group than in the low-risk group in the bladder cancer cohort (Figure 8(a)). The heatmap also demonstrated the differences in terms of pN stage, pT stage, clinical stage, grade, immuneScore, and cluster subtype between the high- and low-risk groups (Figure 8(a), all *p* < 0.01). More specifically, bladder cancer in stage N1–N3 was related to a higher risk score than that in stage N0 (Figure 8(b), *p* = 0.0062). Moreover, the risk score increased when pT stage increased (Figure 8(c), *p*=0.0013). Similarly, the risk score increased as the clinical stage increased (Figure 8(d), *p*=0.00026). The low-grade group had a lower risk score compared with the high-grade group (Figure 8(e), *p*=0.00052). The risk score of the high immuneScore group was significantly higher than that of the low immuneScore group (Figure 8(f), *p*=8.1*e*^−7^). The results also revealed a different risk score between the two clusters (Figure 8(g), *p*=3.9*e*^−16^). These results demonstrated that the risk score was linked to the clinical characteristics in BC.

### 3.7. Risk Score Associated with Immune Infiltration

**Figure 9:**
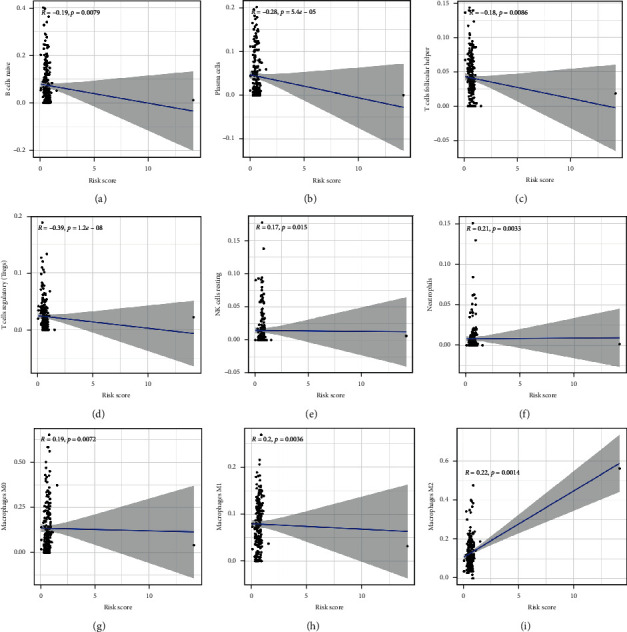
The risk score correlated with immune cell infiltration. The risk score was negatively correlated with the abundance of B cells (a), plasma cells (b), follicular helper T cells (c), and regulatory T cells (d) while it was positively correlated with the abundance of NK resting cells (e), neutrophils (f), M0 macrophages (g), M1 macrophages (h), and M2 macrophages (i).

The risk score negatively correlated with the abundance of naïve B cells (Figure 9(a), *p*=0.0079), plasma cells (Figure 9(b), *p*=5.4*e*^−5^), follicular helper T cells (Figure 9(c), *p*=5.4*e*^−5^), and regulatory T cells (Figure 9(d), *p*=1.2*e*^−8^). A remarkable positive association was obtained between the risk score and the abundance of resting NK cells (Figure 9(e)), neutrophils (Figure 9(f)), M0 macrophages (Figure 9(g)), M1 macrophages (Figure 9(h)), and M2 macrophages (Figure 9(i)) (all *p* < 0.05). We then explored immune checkpoint expression in the high-risk and low-risk groups. As shown in Supplementary Figures 10A–10G, the expression of PD-L1 (*p*=2*e*^−11^), CTLA4 (*p*=4.5*e*^−8^), HAVCR2 (*p*=1*e*^−15^), LAG3 (*p*=1.5*e*^−9^), PDCD1 (*p*=5.7*e*^−7^), PDCDG2 (*p*=2.22*e*^−16^), and TIGIT (*p*=7.6*e*^−8^) in BC tissues was significantly higher than that in normal tissues. We also found a downregulation of SIGLEC15 in BC tissues compared with that in normal tissues (Supplementary Figure 10H, *p*=2.22*e*^−22^). These data indicated that the m6A-related lncRNA prognostic signature was implicated in the bladder cancer immune microenvironment.

## 4. Discussion

m6A methylation is the most common form of mRNA modification and plays a vital role in regulating gene expression at the posttranscriptional level [17]. Abnormal m6A methylation plays a vital role in the progression of cancer by regulating many biological processes, including cell differentiation, immunoreaction, and miRNA editing [7]. Increasingly, researchers have focused on developing lncRNA signatures to evaluate the prognosis of cancer patients [18]. However, limited studies have been performed to study the role of m6A-related lncRNAs in patient prognosis and the immune microenvironment of malignancies, including bladder cancer. In our study, we performed a comprehensive analysis of the expression, prognostic value, and effects on the immune microenvironment of m6A-related lncRNAs in bladder cancer.

We first identified m6A-related lncRNAs by constructing a coexpression network, and a total of 762 m6A-related lncRNAs were obtained. This was followed by univariate Cox regression analysis for the identification of m6A-related prognostic lncRNAs. As a result, a total of 9 m6A-related lncRNAs were significantly related to overall survival outcomes in bladder cancer patients, and the expression of all of these lncRNAs was upregulated or downregulated in bladder cancer tissues compared with that in normal tissues. Thus, these 9 m6A-related prognostic lncRNAs were selected for further analysis. Based on consensus clustering for the 9 m6A-related prognostic lncRNAs, two subtypes (cluster 1 and cluster 2) of bladder cancer were identified. Interestingly, the cluster 1/2 subtype stratification showed a significant correlation with the prognosis and clinical stage of bladder cancer patients and PD-L1 expression. Moreover, we found higher immuneScores, stromalScores, and ESTIMATEScores in cluster 1 compared with those in cluster 2. Interestingly, these results were consistent with the conclusion of the previous study, which revealed that bladder cancer patients with high immuneScore and stromalScore had a poor overall survival rate [19].

The GSEA results revealed that the spliceosome and mTOR signaling pathways were significantly associated with cluster 1. Apoptosis and the JAK-STAT signaling pathway were associated with cluster 2. The JAK-STAT signaling pathway plays a significant role in many biological processes, including cell division, apoptosis, and immune regulation [20, 21]. The mTOR signaling pathway is one of the most investigated therapeutic targets in bladder cancer research [22]. Apoptosis inhibition is an important hallmark of bladder cancer. The above results revealed that cluster 2 bladder cancer patients had a better overall survival outcome than those in cluster 1. Thus, we hypothesized that apoptosis and the JAK-STAT signaling pathway were more active in cluster 2 than in cluster 1.

LASSO Cox analysis was performed to construct the m6A-related lncRNA prognostic signature based on the 9 m6A-related prognostic lncRNAs. Based on the risk scores for the overall survival outcome of each patient calculated by the LASSO algorithm, bladder patients were separated into high- and low-risk groups. We found that bladder cancer patients in the high-risk group had a poor prognosis compared with those in the low-risk group. This signature of 9 m6A-related lncRNAs had a favorable discrimination performance for predicting bladder cancer patient prognosis. This result is consistent with the results of previous studies, which have revealed that lncRNA-related signatures play a vital role in predicting the prognosis of bladder cancer patients. Wang et al. performed a bioinformatics analysis to identify a signature of seven immune-related lncRNAs, which could serve as prognostic biomarkers for bladder cancer [23]. Another prognostic signature based on immune-related lncRNAs can be used to predict the prognosis and immunotherapeutic response of bladder cancer patients [24]. Moreover, an eight-lncRNA signature was suggested as a candidate prognostic biomarker for bladder cancer [25]. In our study, we developed a prognostic signature based on 9 previously unstudied m6A-related prognostic lncRNAs for bladder cancer. Univariate and multivariate Cox regression analyses revealed that the risk score was an independent factor for predicting the prognosis of bladder cancer patients.

We then analyzed the association between the prognostic risk scores and clinical characteristics of bladder cancer patients. We found that patients with bladder cancer in stage N1–N3 and with a low tumor grade had a higher risk score compared with those with N0 and high-grade tumors. The risk score increased as the pT stage and the clinical stage increased. Interestingly, the risk score of the high immuneScore group was significantly higher than that of the low immuneScore group. This finding was consistent with the results of a previous study, which indicated that bladder cancer patients with a high immuneScore had a poor overall survival compared with those with a low immuneScore [26].

The tumor immune microenvironment exerts a vital function in tumorigenesis and cancer progression, and its heterogeneity can influence multiple factors, including patient prognosis and therapeutic response [27, 28]. The results of a previous study indicated that immune cell infiltration can regulate tumor progression and metastasis, thus affecting patient prognosis [29, 30]. Another important finding of our study is that the risk score was correlated with the infiltration levels of certain immune cells, including B cells, plasma cells, follicular helper T cells, regulatory T cells, resting NK cells, neutrophils, M0 macrophages, M1 macrophages, and M2 macrophages. As the risk score increased, the infiltration levels of neutrophils increased. This result was consistent with previous results, which suggested that tumor-infiltrating neutrophils were associated with a high risk of disease recurrence and poor overall survival outcomes [31]. A previous study revealed better overall survival rates in bladder cancer patients with high regulatory T-cell infiltration levels [32]. In our study, we found a negative correlation between regulatory T-cell infiltration levels and the risk score. Bladder cancer patients with a high risk score had poor overall survival. This result was consistent with previous results.

There are some limitations of the current study. First, our results were obtained by analyzing data from the TCGA, and it would be beneficial to verify our findings using the GEO database. The regulatory mechanism of the m6A-related lncRNA prognostic signature warrants further investigation to determine methods to reshape the immune microenvironment and improve precision immunotherapy for bladder cancer.

## 5. Conclusions

In conclusion, this study systematically analyzed the expression, prognostic value, and effects on the immune microenvironment of m6A-related lncRNAs in bladder cancer. By consensus clustering for m6A-related prognostic lncRNAs and construction of a prognostic signature, our study elucidated the important role of m6A-related lncRNAs in patient prognosis and the immune microenvironment in bladder cancer. The results suggest that the m6A-related prognostic lncRNA signature might serve as a crucial mediator of the immune microenvironment in bladder cancer, representing promising therapeutic targets for improving immunotherapeutic efficacy. The findings of our study provided potentially theoretical foundation for future animal and clinical studies about m6A-related lncRNAs as promising therapeutic targets for bladder cancer.

## Figures and Tables

**Table 1:** The clinical characteristics of bladder cancer patients in TCGA cohort.

Clinical characteristics	Number
Gender	
Male	304
Female	108

Age (years, *x* ± *s*)	68.1 ± 10.5

Grade	
High	388
Low	21
Unknown	3

TNM stage	
I	2
II	131
III	141
IV	136
Unknown	2

T	
T0	1
T1	3
T2	120
T3	196
T4	59
Unknown	33

N	
N0	237
N1	47
N2	76
N3	8
Unknown	41

M	
M0	196
M1	11
Unknown	205

**Table 2:** m6A-related lncRNAs with significant prognostic value in bladder cancer identified by Cox regression analysis.

lncRNA	HR	HR.95L	HR.95H	*p* value
PTOV1-AS2	0.884187841	0.82541651	0.947143811	0.000452515
AC116914.2	0.703807678	0.586719475	0.844262494	0.000154644
EHMT2-AS1	0.27801516	0.137558402	0.5618881	0.000362912
AC004148.1	0.831239083	0.747870199	0.92390152	0.000608535
AL136295.2	0.483637966	0.324345308	0.721162527	0.000365815
KCNQ1OT1	2.155118866	1.384412256	3.354880243	0.000672681
AC104564.3	0.595066715	0.437124167	0.810077371	0.000972718
ATP1B3-AS1	1.207264443	1.090085299	1.337039806	0.000299467
AC073534.2	0.573344516	0.41242174	0.797057724	0.000934795

Note: Cox regression analysis was performed with *p* < 0.0001.

## Data Availability

All data generated or analyzed during this study are included in this published article.

## Supplementary Materials

Supplementary Materials Supplementary Figure 1: consensus clustering of m6A-related prognostic lncRNAs. The CDF (A), relative change in area under the CDF curve (B), and tracking plot (C) for *k* = 2–9 in consensus clustering analysis. CDF: cumulative distribution function. Supplementary Figure 2: the correlation between M1 macrophages and M2 macrophages. Supplementary Figure 3: the correlation between immune checkpoints and m6A-related prognostic lncRNAs in bladder cancer; the correlation between m6A-related prognostic lncRNAs and PD-L1 (A), CTLA4 (B), HAVCR2 (C), and LAG3 (D) in bladder cancer. ^*∗*^*p* < 0.05. Supplementary Figure 4: the correlation between immune checkpoints and m6A-related prognostic lncRNAs in bladder cancer; the correlation between m6A-related prognostic lncRNAs and PDCD1 (A), PDCD1LG2 (B), TIGIT (C), and SIGLEC15 (D) in bladder cancer. ^*∗*^*p* < 0.05. Supplementary Figure 5: gene set enrichment analysis (GSEA) in cluster 1 of bladder cancer. GSEA showed that spliceosome, mTOR signaling pathway, and Notch signaling pathway were significantly associated with cluster 1. Supplementary Figure 6: Gene set enrichment analysis (GSEA) in cluster 2 of bladder cancer. GSEA showed that apoptosis, chemokine signaling pathway, Toll-like receptor signaling pathway, and JAK-STAT signaling pathway were enriched in cluster 2. Supplementary Figure 7: survival curve of the high-/low-risk group in different subtypes of bladder cancer patients. Overall survival curve revealed a poor survival probability in high-risk group patients with age >50 years (A), male and female patients (B), and patients with high tumor grade (C). Supplementary Figure 8: survival curve of the high-/low-risk group in different subtypes of bladder cancer patients. Overall survival curve revealed a poor survival probability in high-risk group patients with T3-4 stage (A), M0 (B), and N0 (C). Supplementary Figure 9: survival curve of the high-/low-risk group in bladder cancer patients with different clinical stages. Overall survival curve revealed a poor survival probability in the high-risk group of patients with clinical stage III-IV. Supplementary Figure 10: the expression of immune checkpoints in high-risk and low-risk groups of bladder cancer patients. Bladder cancer patients in the high-risk group had a higher expression of PD-L1 (A), CTLA4 (B), HAVCR2 (C), LAG3 (D), PDCD1 (E), PDCD1LG2 (F), and TIGIT (G) and a lower expression of SIGLEC15 (H) compared with those in the low-risk group.

## References

[1] Quan J., Pan X., Zhao L. (2018). LncRNA as a diagnostic and prognostic biomarker in bladder cancer: a systematic review and meta-analysis. *OncoTargets and Therapy*.

[2] Grayson M. (2017). Bladder cancer. *Nature*.

[3] Ghandour R., Singla N., Lotan Y. (2019). Treatment options and outcomes in nonmetastatic muscle invasive bladder cancer. *Trends in Cancer*.

[4] Fujii Y. (2018). Prediction models for progression of non-muscle-invasive bladder cancer: a review. *International Journal of Urology*.

[5] Tu Z., Wu L., Wang P. (2020). N6-methylandenosine-related lncRNAs are potential biomarkers for predicting the overall survival of lower-grade glioma patients. *Frontiers in Cell and Developmental Biology*.

[6] He L., Li H., Wu A., Peng Y., Shu G., Yin G. (2019). Functions of N6-methyladenosine and its role in cancer. *Molecular Cancer*.

[7] Yi L., Wu G., Guo L., Zou X., Huang P. (2020). Comprehensive analysis of the PD-L1 and immune infiltrates of m6A RNA methylation regulators in head and neck squamous cell carcinoma. *Molecular Therapy-Nucleic Acids*.

[8] Liu T., Li C., Jin L., Li C., Wang L. (2019). The prognostic value of m6A RNA methylation regulators in colon adenocarcinoma. *Medical Science Monitor*.

[9] Ruan J., Wang S., Wang J. (2020). Mechanism and regulation of pyroptosis-mediated in cancer cell death. *Chemico-Biological Interactions*.

[10] Chen M., Nie Z. Y., Wen X. H., Gao Y. H., Cao H., Zhang S. F. (2019). m6A RNA methylation regulators can contribute to malignant progression and impact the prognosis of bladder cancer. *Bioscience Reports*.

[11] Bhan A., Soleimani M., Mandal S. S. (2017). Long noncoding RNA and cancer: a new paradigm. *Cancer Research*.

[12] Li Z., Hong S., Liu Z. (2018). LncRNA LINC00641 predicts prognosis and inhibits bladder cancer progression through miR-197-3p/KLF10/PTEN/PI3K/AKT cascade. *Biochemical and Biophysical Research Communications*.

[13] Zhang C., Wang W., Lin J., Xiao J., Tian Y. (2019). lncRNA CCAT1 promotes bladder cancer cell proliferation, migration and invasion. *International Brazilian Journal of Urology*.

[14] Tomczak K., Czerwińska P., Wiznerowicz M. (2015). The Cancer Genome Atlas (TCGA): an immeasurable source of knowledge. *Contemporary Oncology (Poznan, Poland)*.

[15] Jin Y., Wang Z., He D. (2021). Analysis of m6A-related signatures in the tumor immune microenvironment and identification of clinical prognostic regulators in adrenocortical carcinoma. *Frontiers in Immunology*.

[16] Chen X.-Y., Zhang J., Zhu J.-S. (2019). The role of m6A RNA methylation in human cancer. *Molecular Cancer*.

[17] He C. (2010). Grand challenge commentary: RNA epigenetics?. *Nature Chemical Biology*.

[18] Zhu X., Tian X., Yu C. (2016). A long non-coding RNA signature to improve prognosis prediction of gastric cancer. *Molecular Cancer*.

[19] Li F., Guo H., Wang Y., Liu B., Zhou H. (2020). Profiles of tumor-infiltrating immune cells and prognostic genes associated with the microenvironment of bladder cancer. *International Immunopharmacology*.

[20] Hindupur S. V., Schmid S. C., Koch J. A. (2020). STAT3/5 inhibitors suppress proliferation in bladder cancer and enhance oncolytic adenovirus therapy. *International Journal of Molecular Sciences*.

[21] Li Y., Shan Z., Liu C. (2017). MicroRNA-294 promotes cellular proliferation and motility through the PI3K/AKT and JAK/STAT pathways by upregulation of NRAS in bladder cancer. *Biochemistry (Moscow)*.

[22] Sathe A., Nawroth R. (2018). Targeting the PI3K/AKT/mTOR pathway in bladder cancer. *Urothelial Carcinoma*.

[23] Wang J., Shen C., Dong D., Zhong X., Wang Y., Yang X. (2021). Identification and verification of an immune-related lncRNA signature for predicting the prognosis of patients with bladder cancer. *International Immunopharmacology*.

[24] Wu Y., Zhang L., He S. (2020). Identification of immune-related LncRNA for predicting prognosis and immunotherapeutic response in bladder cancer. *Aging*.

[25] Lian P., Wang Q., Zhao Y. (2019). An eight-long non-coding RNA signature as a candidate prognostic biomarker for bladder cancer. *Aging*.

[26] Peng Y. L., Wu Z. S., Lu H. M. (2020). Prognostic significance of tumor-infiltrating immune cells in muscle-invasive bladder cancer. *American Journal of Translational Research*.

[27] Fridman W. H., Pagès F., Sautès-Fridman C., Galon J. (2012). The immune contexture in human tumours: impact on clinical outcome. *Nature Reviews Cancer*.

[28] Hui L., Chen Y. (2015). Tumor microenvironment: sanctuary of the devil. *Cancer Letters*.

[29] Yang S., Liu T., Nan H. (2019). Comprehensive analysis of prognostic immune-related genes in the tumor microenvironment of cutaneous melanoma. *Journal of Cellular Physiology*.

[30] Bremnes R. M., Busund L.-T., Kilvær T. L. (2016). The role of tumor-infiltrating lymphocytes in development, progression, and prognosis of non-small cell lung cancer. *Journal of Thoracic Oncology*.

[31] Xu Q., Wang C., Yuan X., Feng Z., Han Z. (2017). Prognostic value of tumor-infiltrating lymphocytes for patients with head and neck squamous cell carcinoma. *Translational Oncology*.

[32] Wu Z., Zhu K., Liu Q. (2020). Profiles of immune infiltration in bladder cancer and its clinical significance: an integrative genomic analysis. *International Journal of Medical Sciences*.

