# Supplementary material for: m6A-Related lncRNA to Develop Prognostic Signature and Predict the Immune Landscape in Bladder Cancer
Source: J Oncol. 2021 Jul 24;2021:7488188. doi: 10.1155/2021/7488188 (PMC8328735; doi:10.1155/2021/7488188)
Supplement: Supplementary Materials — Supplementary Figure 1: consensus clustering of m6A-related prognostic lncRNAs. The CDF (A), relative change in area under the CDF curve (B), and tracking plot (C) for k = 2–9 in consensus clustering analysis. CDF: cumulative distribution function. Supplementary Figure 2: the correlation between M1 macrophages and M2 macrophages. Supplementary Figure 3: the correlation between immune checkpoints and m6A-related prognostic lncRNAs in bladder cancer; the correlation between m6A-related prognostic lncRNAs and PD-L1 (A), CTLA4 (B), HAVCR2 (C), and LAG3 (D) in bladder cancer. ∗p < 0.05. Supplementary Figure 4: the correlation between immune checkpoints and m6A-related prognostic lncRNAs in bladder cancer; the correlation between m6A-related prognostic lncRNAs and PDCD1 (A), PDCD1LG2 (B), TIGIT (C), and SIGLEC15 (D) in bladder cancer. ∗p < 0.05. Supplementary Figure 5: gene set enrichment analysis (GSEA) in cluster 1 of bladder cancer. GSEA showed that spliceosome, mTOR signaling pathway, and Notch signaling pathway were significantly associated with cluster 1. Supplementary Figure 6: Gene set enrichment analysis (GSEA) in cluster 2 of bladder cancer. GSEA showed that apoptosis, chemokine signaling pathway, Toll-like receptor signaling pathway, and JAK-STAT signaling pathway were enriched in cluster 2. Supplementary Figure 7: survival curve of the high-/low-risk group in different subtypes of bladder cancer patients. Overall survival curve revealed a poor survival probability in high-risk group patients with age >50 years (A), male and female patients (B), and patients with high tumor grade (C). Supplementary Figure 8: survival curve of the high-/low-risk group in different subtypes of bladder cancer patients. Overall survival curve revealed a poor survival probability in high-risk group patients with T3-4 stage (A), M0 (B), and N0 (C). Supplementary Figure 9: survival curve of the high-/low-risk group in bladder cancer patients with different clinical stages. Overall sur [file 7488188.f1.zip › 7488188.f1/Supplementary Figure 6.pdf]

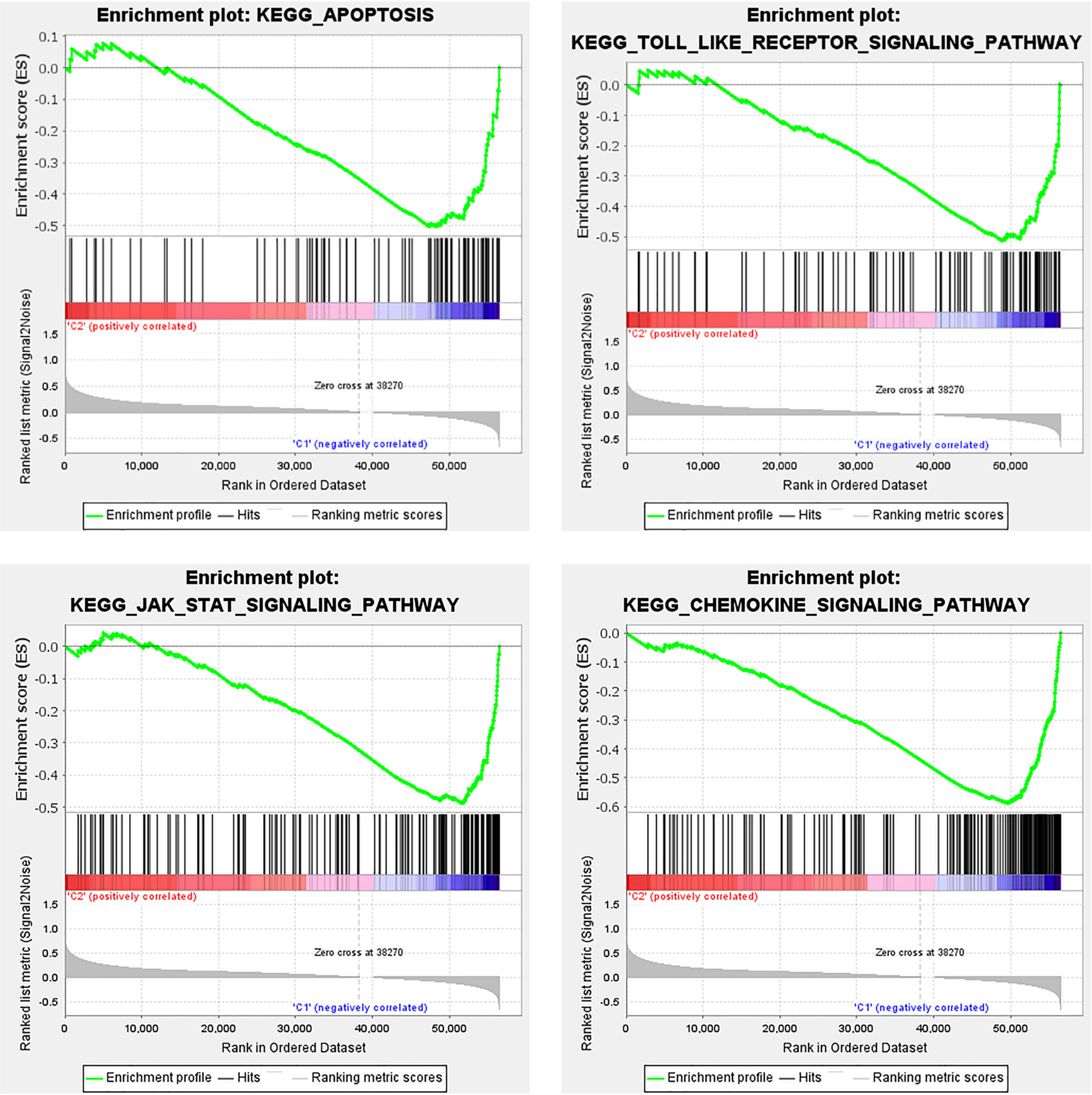
Enrichment plot: KEGG_APOPTOSIS
Enrichment score (ES)
0.1
0.0
-0.1
-0.2
-0.3
-0.4
-0.5
Ranked list metric (Signal2Noise)
'C2' (positively correlated)
1.5
1.0
0.5
0.0
-0.5
Zero cross at 38270
'C1' (negatively correlated)
0
10,000
20,000
30,000
40,000
50,000
Rank in Ordered Dataset
Enrichment profile
Hits
Ranking metric scores
Enrichment plot:
KEGG_TOLL_LIKE_RECEPTOR_SIGNALING_PATHWAY
Enrichment score (ES)
0.0
-0.1
-0.2
-0.3
-0.4
-0.5
Ranked list metric (Signal2Noise)
'C2' (positively correlated)
1.5
1.0
0.5
0.0
-0.5
Zero cross at 38270
'C1' (negatively correlated)
0
10,000
20,000
30,000
40,000
50,000
Rank in Ordered Dataset
Enrichment profile
Hits
Ranking metric scores
Enrichment plot:
KEGG_JAK_STAT_SIGNALING_PATHWAY
Enrichment score (ES)
0.0
-0.1
-0.2
-0.3
-0.4
-0.5
Ranked list metric (Signal2Noise)
'C2' (positively correlated)
1.5
1.0
0.5
0.0
-0.5
Zero cross at 38270
'C1' (negatively correlated)
0
10,000
20,000
30,000
40,000
50,000
Rank in Ordered Dataset
Enrichment profile
Hits
Ranking metric scores
Enrichment plot:
KEGG_CHEMOKINE_SIGNALING_PATHWAY
Enrichment score (ES)
0.0
-0.1
-0.2
-0.3
-0.4
-0.5
-0.6
Ranked list metric (Signal2Noise)
'C2' (positively correlated)
1.5
1.0
0.5
0.0
-0.5
Zero cross at 38270
'C1' (negatively correlated)
0
10,000
20,000
30,000
40,000
50,000
Rank in Ordered Dataset
Enrichment profile
Hits
Ranking metric scores
