# Supplementary figures and images for: m6A-Related lncRNA to Develop Prognostic Signature and Predict the Immune Landscape in Bladder Cancer
Source: J Oncol. 2021 Jul 24;2021:7488188. doi: 10.1155/2021/7488188 (PMC8328735; doi:10.1155/2021/7488188)

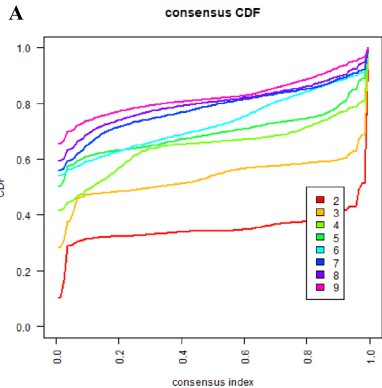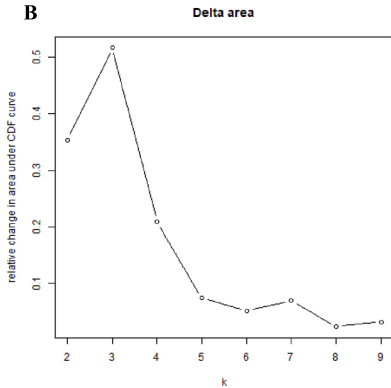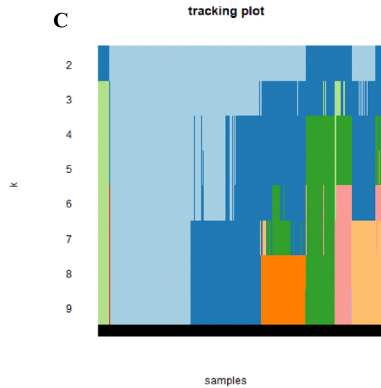

Supplement: Supplementary Materials — Supplementary Figure 1: consensus clustering of m6A-related prognostic lncRNAs. The CDF (A), relative change in area under the CDF curve (B), and tracking plot (C) for k = 2–9 in consensus clustering analysis. CDF: cumulative distribution function. Supplementary Figure 2: the correlation between M1 macrophages and M2 macrophages. Supplementary Figure 3: the correlation between immune checkpoints and m6A-related prognostic lncRNAs in bladder cancer; the correlation between m6A-related prognostic lncRNAs and PD-L1 (A), CTLA4 (B), HAVCR2 (C), and LAG3 (D) in bladder cancer. ∗p < 0.05. Supplementary Figure 4: the correlation between immune checkpoints and m6A-related prognostic lncRNAs in bladder cancer; the correlation between m6A-related prognostic lncRNAs and PDCD1 (A), PDCD1LG2 (B), TIGIT (C), and SIGLEC15 (D) in bladder cancer. ∗p < 0.05. Supplementary Figure 5: gene set enrichment analysis (GSEA) in cluster 1 of bladder cancer. GSEA showed that spliceosome, mTOR signaling pathway, and Notch signaling pathway were significantly associated with cluster 1. Supplementary Figure 6: Gene set enrichment analysis (GSEA) in cluster 2 of bladder cancer. GSEA showed that apoptosis, chemokine signaling pathway, Toll-like receptor signaling pathway, and JAK-STAT signaling pathway were enriched in cluster 2. Supplementary Figure 7: survival curve of the high-/low-risk group in different subtypes of bladder cancer patients. Overall survival curve revealed a poor survival probability in high-risk group patients with age >50 years (A), male and female patients (B), and patients with high tumor grade (C). Supplementary Figure 8: survival curve of the high-/low-risk group in different subtypes of bladder cancer patients. Overall survival curve revealed a poor survival probability in high-risk group patients with T3-4 stage (A), M0 (B), and N0 (C). Supplementary Figure 9: survival curve of the high-/low-risk group in bladder cancer patients with different clinical stages. Overall sur [file 7488188.f1.zip › 7488188.f1/Supplementary Figure 1.pdf]

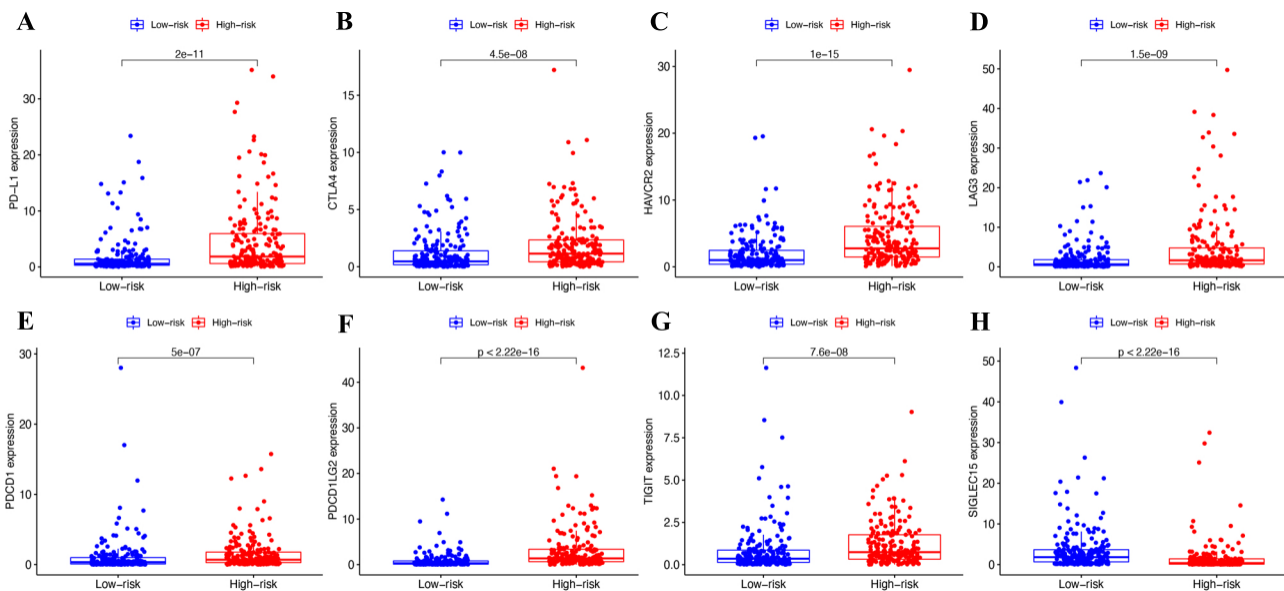

Supplement: Supplementary Materials — Supplementary Figure 1: consensus clustering of m6A-related prognostic lncRNAs. The CDF (A), relative change in area under the CDF curve (B), and tracking plot (C) for k = 2–9 in consensus clustering analysis. CDF: cumulative distribution function. Supplementary Figure 2: the correlation between M1 macrophages and M2 macrophages. Supplementary Figure 3: the correlation between immune checkpoints and m6A-related prognostic lncRNAs in bladder cancer; the correlation between m6A-related prognostic lncRNAs and PD-L1 (A), CTLA4 (B), HAVCR2 (C), and LAG3 (D) in bladder cancer. ∗p < 0.05. Supplementary Figure 4: the correlation between immune checkpoints and m6A-related prognostic lncRNAs in bladder cancer; the correlation between m6A-related prognostic lncRNAs and PDCD1 (A), PDCD1LG2 (B), TIGIT (C), and SIGLEC15 (D) in bladder cancer. ∗p < 0.05. Supplementary Figure 5: gene set enrichment analysis (GSEA) in cluster 1 of bladder cancer. GSEA showed that spliceosome, mTOR signaling pathway, and Notch signaling pathway were significantly associated with cluster 1. Supplementary Figure 6: Gene set enrichment analysis (GSEA) in cluster 2 of bladder cancer. GSEA showed that apoptosis, chemokine signaling pathway, Toll-like receptor signaling pathway, and JAK-STAT signaling pathway were enriched in cluster 2. Supplementary Figure 7: survival curve of the high-/low-risk group in different subtypes of bladder cancer patients. Overall survival curve revealed a poor survival probability in high-risk group patients with age >50 years (A), male and female patients (B), and patients with high tumor grade (C). Supplementary Figure 8: survival curve of the high-/low-risk group in different subtypes of bladder cancer patients. Overall survival curve revealed a poor survival probability in high-risk group patients with T3-4 stage (A), M0 (B), and N0 (C). Supplementary Figure 9: survival curve of the high-/low-risk group in bladder cancer patients with different clinical stages. Overall sur [file 7488188.f1.zip › 7488188.f1/Supplementary Figure 10.pdf]

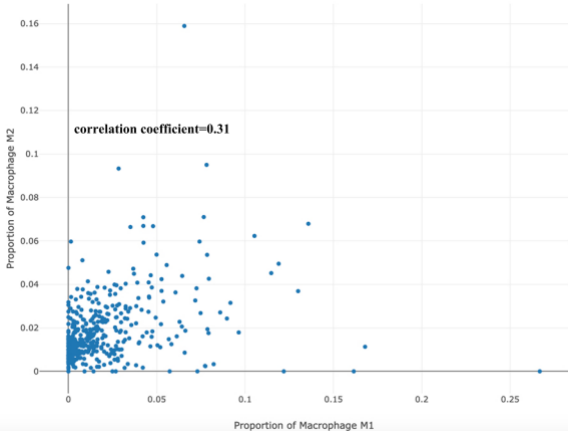

Supplement: Supplementary Materials — Supplementary Figure 1: consensus clustering of m6A-related prognostic lncRNAs. The CDF (A), relative change in area under the CDF curve (B), and tracking plot (C) for k = 2–9 in consensus clustering analysis. CDF: cumulative distribution function. Supplementary Figure 2: the correlation between M1 macrophages and M2 macrophages. Supplementary Figure 3: the correlation between immune checkpoints and m6A-related prognostic lncRNAs in bladder cancer; the correlation between m6A-related prognostic lncRNAs and PD-L1 (A), CTLA4 (B), HAVCR2 (C), and LAG3 (D) in bladder cancer. ∗p < 0.05. Supplementary Figure 4: the correlation between immune checkpoints and m6A-related prognostic lncRNAs in bladder cancer; the correlation between m6A-related prognostic lncRNAs and PDCD1 (A), PDCD1LG2 (B), TIGIT (C), and SIGLEC15 (D) in bladder cancer. ∗p < 0.05. Supplementary Figure 5: gene set enrichment analysis (GSEA) in cluster 1 of bladder cancer. GSEA showed that spliceosome, mTOR signaling pathway, and Notch signaling pathway were significantly associated with cluster 1. Supplementary Figure 6: Gene set enrichment analysis (GSEA) in cluster 2 of bladder cancer. GSEA showed that apoptosis, chemokine signaling pathway, Toll-like receptor signaling pathway, and JAK-STAT signaling pathway were enriched in cluster 2. Supplementary Figure 7: survival curve of the high-/low-risk group in different subtypes of bladder cancer patients. Overall survival curve revealed a poor survival probability in high-risk group patients with age >50 years (A), male and female patients (B), and patients with high tumor grade (C). Supplementary Figure 8: survival curve of the high-/low-risk group in different subtypes of bladder cancer patients. Overall survival curve revealed a poor survival probability in high-risk group patients with T3-4 stage (A), M0 (B), and N0 (C). Supplementary Figure 9: survival curve of the high-/low-risk group in bladder cancer patients with different clinical stages. Overall sur [file 7488188.f1.zip › 7488188.f1/Supplementary Figure 2.pdf]

**A**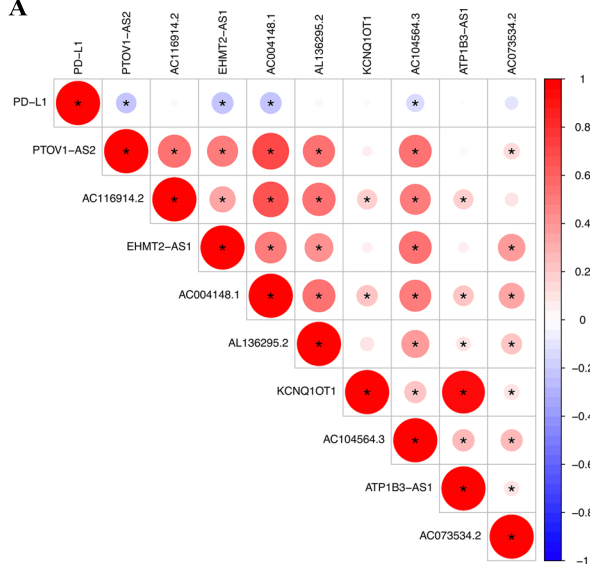**B**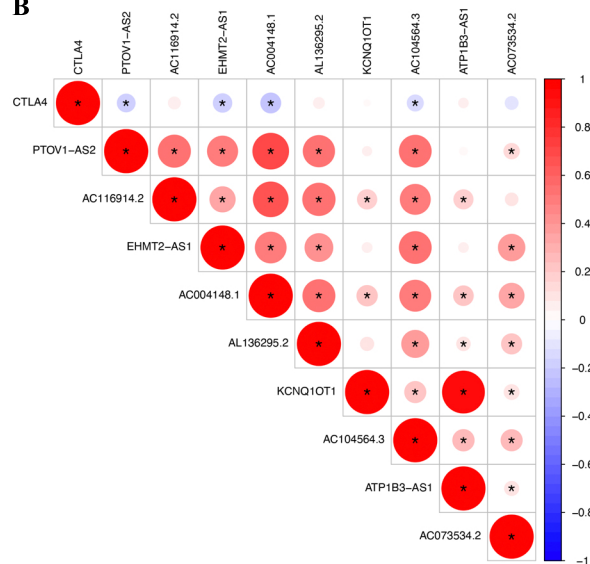**C**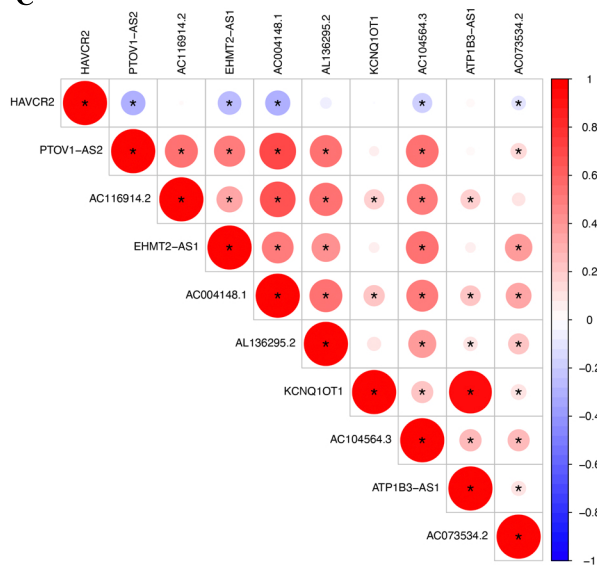**D**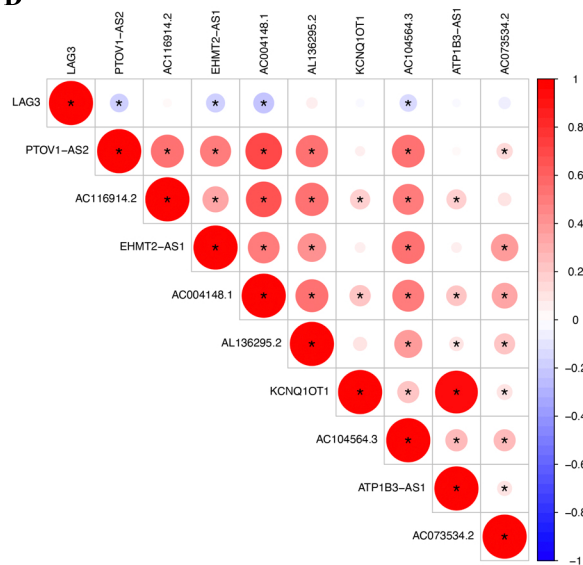

Supplement: Supplementary Materials — Supplementary Figure 1: consensus clustering of m6A-related prognostic lncRNAs. The CDF (A), relative change in area under the CDF curve (B), and tracking plot (C) for k = 2–9 in consensus clustering analysis. CDF: cumulative distribution function. Supplementary Figure 2: the correlation between M1 macrophages and M2 macrophages. Supplementary Figure 3: the correlation between immune checkpoints and m6A-related prognostic lncRNAs in bladder cancer; the correlation between m6A-related prognostic lncRNAs and PD-L1 (A), CTLA4 (B), HAVCR2 (C), and LAG3 (D) in bladder cancer. ∗p < 0.05. Supplementary Figure 4: the correlation between immune checkpoints and m6A-related prognostic lncRNAs in bladder cancer; the correlation between m6A-related prognostic lncRNAs and PDCD1 (A), PDCD1LG2 (B), TIGIT (C), and SIGLEC15 (D) in bladder cancer. ∗p < 0.05. Supplementary Figure 5: gene set enrichment analysis (GSEA) in cluster 1 of bladder cancer. GSEA showed that spliceosome, mTOR signaling pathway, and Notch signaling pathway were significantly associated with cluster 1. Supplementary Figure 6: Gene set enrichment analysis (GSEA) in cluster 2 of bladder cancer. GSEA showed that apoptosis, chemokine signaling pathway, Toll-like receptor signaling pathway, and JAK-STAT signaling pathway were enriched in cluster 2. Supplementary Figure 7: survival curve of the high-/low-risk group in different subtypes of bladder cancer patients. Overall survival curve revealed a poor survival probability in high-risk group patients with age >50 years (A), male and female patients (B), and patients with high tumor grade (C). Supplementary Figure 8: survival curve of the high-/low-risk group in different subtypes of bladder cancer patients. Overall survival curve revealed a poor survival probability in high-risk group patients with T3-4 stage (A), M0 (B), and N0 (C). Supplementary Figure 9: survival curve of the high-/low-risk group in bladder cancer patients with different clinical stages. Overall sur [file 7488188.f1.zip › 7488188.f1/Supplementary Figure 3.pdf]

A

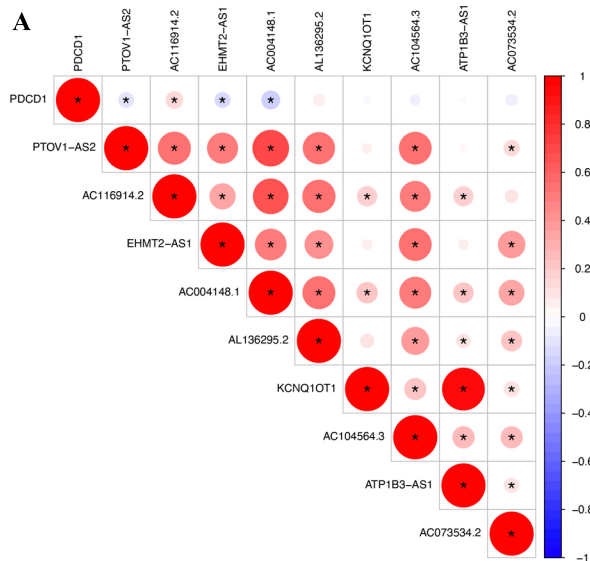**B**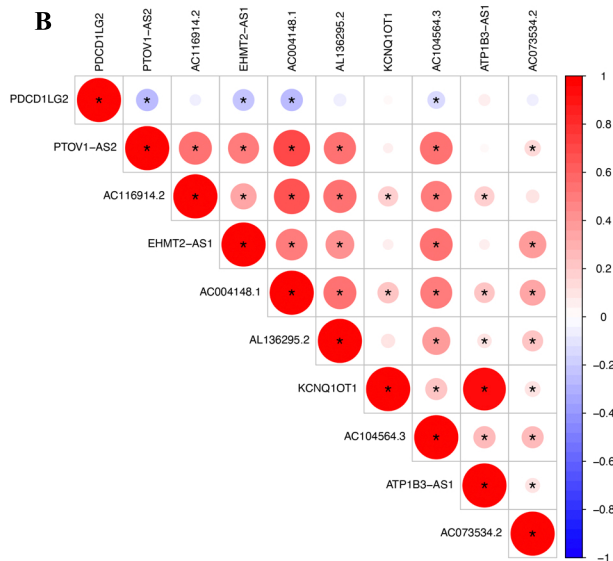

C

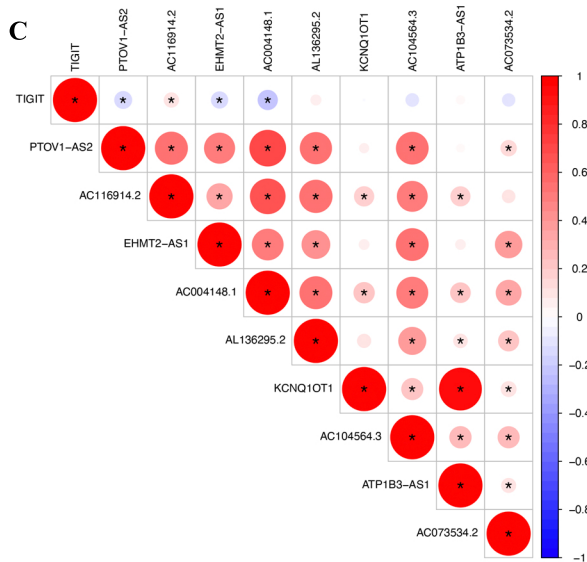

D

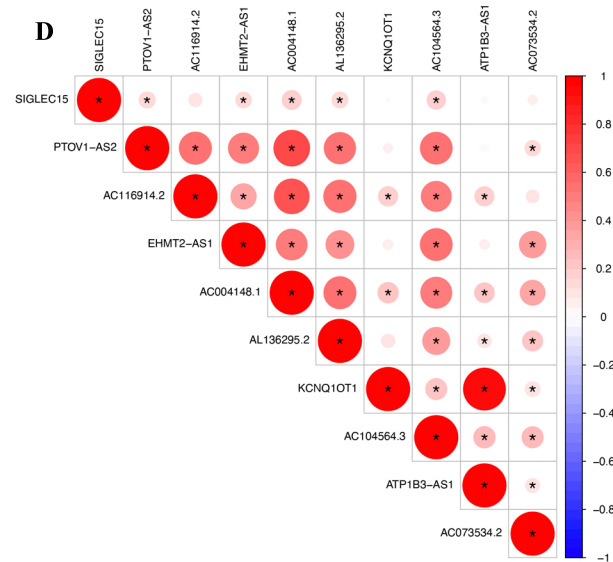

Supplement: Supplementary Materials — Supplementary Figure 1: consensus clustering of m6A-related prognostic lncRNAs. The CDF (A), relative change in area under the CDF curve (B), and tracking plot (C) for k = 2–9 in consensus clustering analysis. CDF: cumulative distribution function. Supplementary Figure 2: the correlation between M1 macrophages and M2 macrophages. Supplementary Figure 3: the correlation between immune checkpoints and m6A-related prognostic lncRNAs in bladder cancer; the correlation between m6A-related prognostic lncRNAs and PD-L1 (A), CTLA4 (B), HAVCR2 (C), and LAG3 (D) in bladder cancer. ∗p < 0.05. Supplementary Figure 4: the correlation between immune checkpoints and m6A-related prognostic lncRNAs in bladder cancer; the correlation between m6A-related prognostic lncRNAs and PDCD1 (A), PDCD1LG2 (B), TIGIT (C), and SIGLEC15 (D) in bladder cancer. ∗p < 0.05. Supplementary Figure 5: gene set enrichment analysis (GSEA) in cluster 1 of bladder cancer. GSEA showed that spliceosome, mTOR signaling pathway, and Notch signaling pathway were significantly associated with cluster 1. Supplementary Figure 6: Gene set enrichment analysis (GSEA) in cluster 2 of bladder cancer. GSEA showed that apoptosis, chemokine signaling pathway, Toll-like receptor signaling pathway, and JAK-STAT signaling pathway were enriched in cluster 2. Supplementary Figure 7: survival curve of the high-/low-risk group in different subtypes of bladder cancer patients. Overall survival curve revealed a poor survival probability in high-risk group patients with age >50 years (A), male and female patients (B), and patients with high tumor grade (C). Supplementary Figure 8: survival curve of the high-/low-risk group in different subtypes of bladder cancer patients. Overall survival curve revealed a poor survival probability in high-risk group patients with T3-4 stage (A), M0 (B), and N0 (C). Supplementary Figure 9: survival curve of the high-/low-risk group in bladder cancer patients with different clinical stages. Overall sur [file 7488188.f1.zip › 7488188.f1/Supplementary Figure 4.pdf]

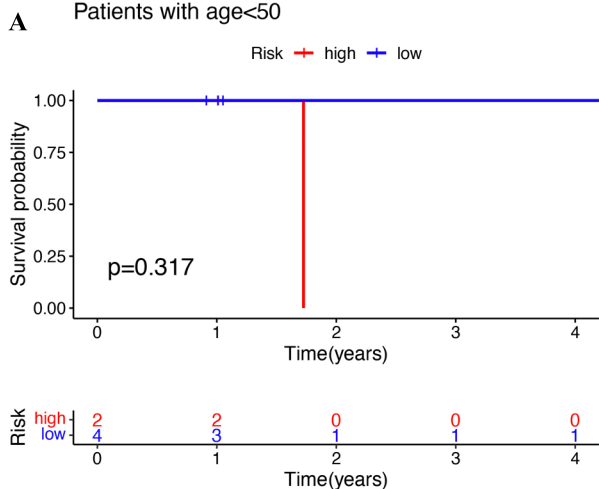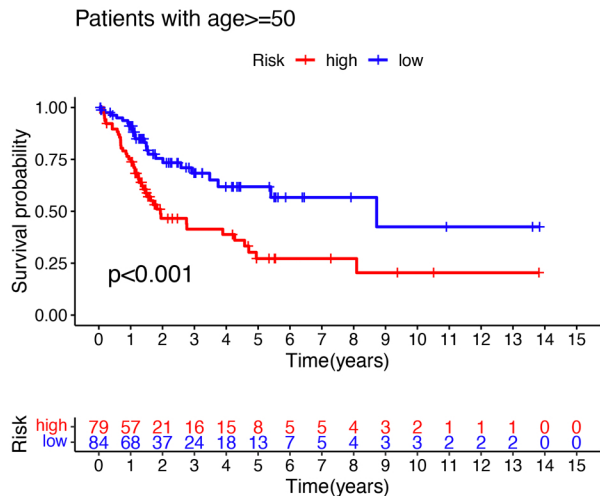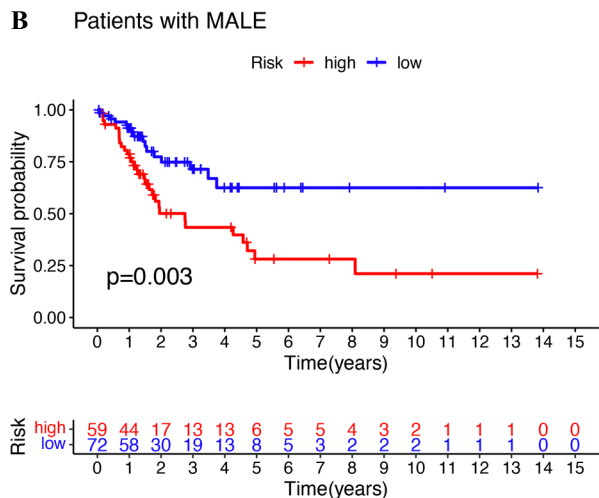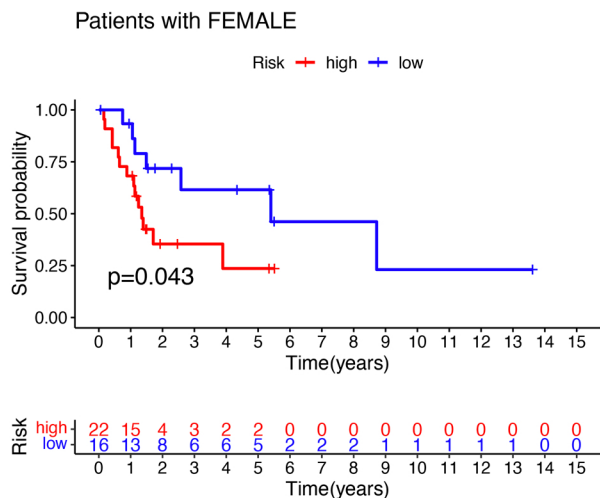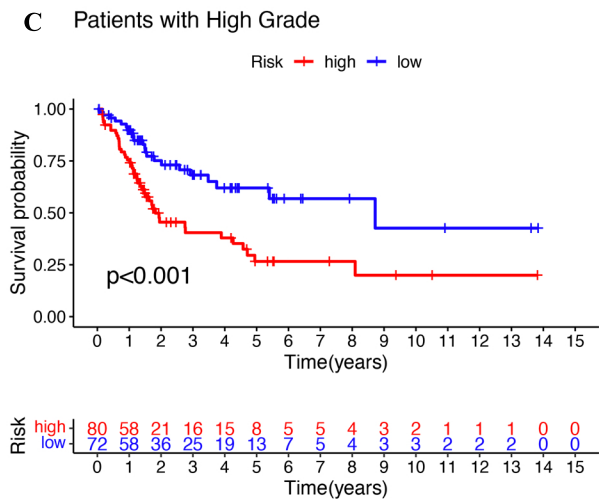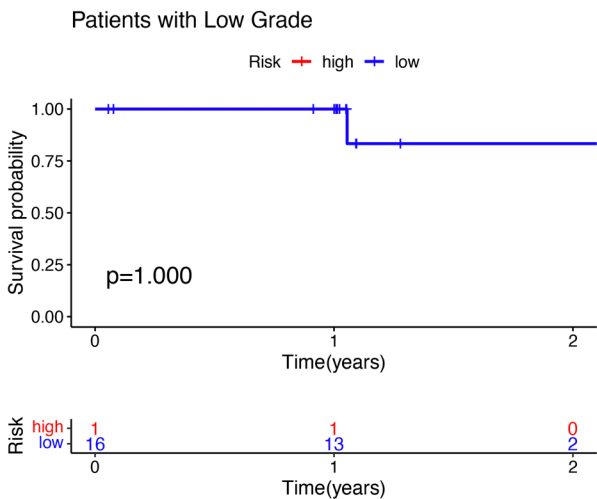

Supplement: Supplementary Materials — Supplementary Figure 1: consensus clustering of m6A-related prognostic lncRNAs. The CDF (A), relative change in area under the CDF curve (B), and tracking plot (C) for k = 2–9 in consensus clustering analysis. CDF: cumulative distribution function. Supplementary Figure 2: the correlation between M1 macrophages and M2 macrophages. Supplementary Figure 3: the correlation between immune checkpoints and m6A-related prognostic lncRNAs in bladder cancer; the correlation between m6A-related prognostic lncRNAs and PD-L1 (A), CTLA4 (B), HAVCR2 (C), and LAG3 (D) in bladder cancer. ∗p < 0.05. Supplementary Figure 4: the correlation between immune checkpoints and m6A-related prognostic lncRNAs in bladder cancer; the correlation between m6A-related prognostic lncRNAs and PDCD1 (A), PDCD1LG2 (B), TIGIT (C), and SIGLEC15 (D) in bladder cancer. ∗p < 0.05. Supplementary Figure 5: gene set enrichment analysis (GSEA) in cluster 1 of bladder cancer. GSEA showed that spliceosome, mTOR signaling pathway, and Notch signaling pathway were significantly associated with cluster 1. Supplementary Figure 6: Gene set enrichment analysis (GSEA) in cluster 2 of bladder cancer. GSEA showed that apoptosis, chemokine signaling pathway, Toll-like receptor signaling pathway, and JAK-STAT signaling pathway were enriched in cluster 2. Supplementary Figure 7: survival curve of the high-/low-risk group in different subtypes of bladder cancer patients. Overall survival curve revealed a poor survival probability in high-risk group patients with age >50 years (A), male and female patients (B), and patients with high tumor grade (C). Supplementary Figure 8: survival curve of the high-/low-risk group in different subtypes of bladder cancer patients. Overall survival curve revealed a poor survival probability in high-risk group patients with T3-4 stage (A), M0 (B), and N0 (C). Supplementary Figure 9: survival curve of the high-/low-risk group in bladder cancer patients with different clinical stages. Overall sur [file 7488188.f1.zip › 7488188.f1/Supplementary Figure 7.pdf]

## A

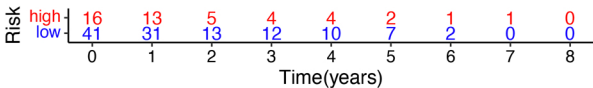

### Patients with T3-4

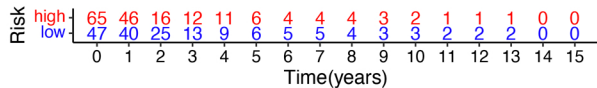

# B

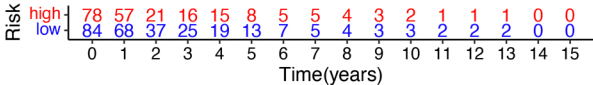

## Patients with M1

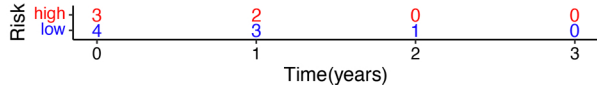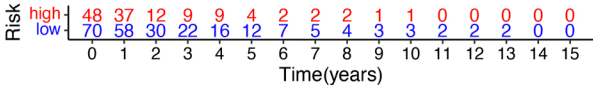

### Patients with N1–3

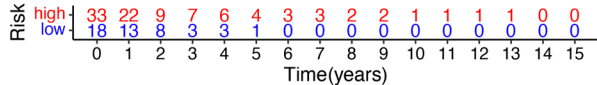

Supplement: Supplementary Materials — Supplementary Figure 1: consensus clustering of m6A-related prognostic lncRNAs. The CDF (A), relative change in area under the CDF curve (B), and tracking plot (C) for k = 2–9 in consensus clustering analysis. CDF: cumulative distribution function. Supplementary Figure 2: the correlation between M1 macrophages and M2 macrophages. Supplementary Figure 3: the correlation between immune checkpoints and m6A-related prognostic lncRNAs in bladder cancer; the correlation between m6A-related prognostic lncRNAs and PD-L1 (A), CTLA4 (B), HAVCR2 (C), and LAG3 (D) in bladder cancer. ∗p < 0.05. Supplementary Figure 4: the correlation between immune checkpoints and m6A-related prognostic lncRNAs in bladder cancer; the correlation between m6A-related prognostic lncRNAs and PDCD1 (A), PDCD1LG2 (B), TIGIT (C), and SIGLEC15 (D) in bladder cancer. ∗p < 0.05. Supplementary Figure 5: gene set enrichment analysis (GSEA) in cluster 1 of bladder cancer. GSEA showed that spliceosome, mTOR signaling pathway, and Notch signaling pathway were significantly associated with cluster 1. Supplementary Figure 6: Gene set enrichment analysis (GSEA) in cluster 2 of bladder cancer. GSEA showed that apoptosis, chemokine signaling pathway, Toll-like receptor signaling pathway, and JAK-STAT signaling pathway were enriched in cluster 2. Supplementary Figure 7: survival curve of the high-/low-risk group in different subtypes of bladder cancer patients. Overall survival curve revealed a poor survival probability in high-risk group patients with age >50 years (A), male and female patients (B), and patients with high tumor grade (C). Supplementary Figure 8: survival curve of the high-/low-risk group in different subtypes of bladder cancer patients. Overall survival curve revealed a poor survival probability in high-risk group patients with T3-4 stage (A), M0 (B), and N0 (C). Supplementary Figure 9: survival curve of the high-/low-risk group in bladder cancer patients with different clinical stages. Overall sur [file 7488188.f1.zip › 7488188.f1/Supplementary Figure 8.pdf]

# Patients with Stage I–II

Risk + high + low

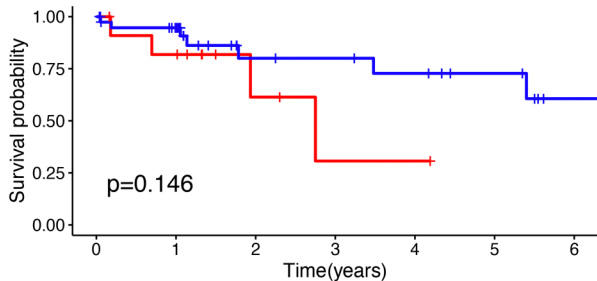

# Patients with Stage III–IV

Risk + high + low

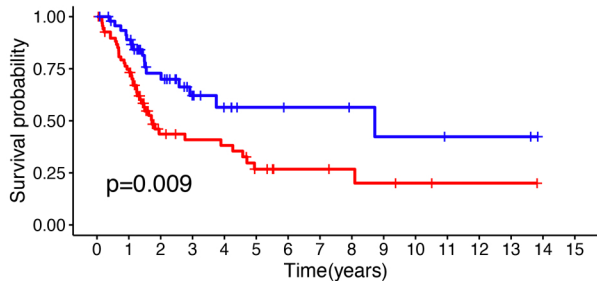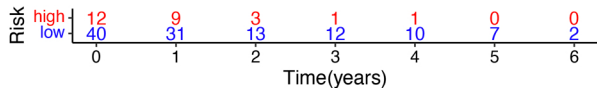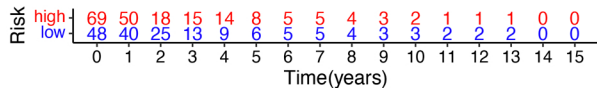

Supplement: Supplementary Materials — Supplementary Figure 1: consensus clustering of m6A-related prognostic lncRNAs. The CDF (A), relative change in area under the CDF curve (B), and tracking plot (C) for k = 2–9 in consensus clustering analysis. CDF: cumulative distribution function. Supplementary Figure 2: the correlation between M1 macrophages and M2 macrophages. Supplementary Figure 3: the correlation between immune checkpoints and m6A-related prognostic lncRNAs in bladder cancer; the correlation between m6A-related prognostic lncRNAs and PD-L1 (A), CTLA4 (B), HAVCR2 (C), and LAG3 (D) in bladder cancer. ∗p < 0.05. Supplementary Figure 4: the correlation between immune checkpoints and m6A-related prognostic lncRNAs in bladder cancer; the correlation between m6A-related prognostic lncRNAs and PDCD1 (A), PDCD1LG2 (B), TIGIT (C), and SIGLEC15 (D) in bladder cancer. ∗p < 0.05. Supplementary Figure 5: gene set enrichment analysis (GSEA) in cluster 1 of bladder cancer. GSEA showed that spliceosome, mTOR signaling pathway, and Notch signaling pathway were significantly associated with cluster 1. Supplementary Figure 6: Gene set enrichment analysis (GSEA) in cluster 2 of bladder cancer. GSEA showed that apoptosis, chemokine signaling pathway, Toll-like receptor signaling pathway, and JAK-STAT signaling pathway were enriched in cluster 2. Supplementary Figure 7: survival curve of the high-/low-risk group in different subtypes of bladder cancer patients. Overall survival curve revealed a poor survival probability in high-risk group patients with age >50 years (A), male and female patients (B), and patients with high tumor grade (C). Supplementary Figure 8: survival curve of the high-/low-risk group in different subtypes of bladder cancer patients. Overall survival curve revealed a poor survival probability in high-risk group patients with T3-4 stage (A), M0 (B), and N0 (C). Supplementary Figure 9: survival curve of the high-/low-risk group in bladder cancer patients with different clinical stages. Overall sur [file 7488188.f1.zip › 7488188.f1/Supplementary Figure 9.pdf]
